# Evaluation of the alkaline water effect on the salivary bacteria of children: preliminary randomized trial

**DOI:** 10.1038/s41598-026-57377-6

**Published:** 2026-06-17

**Authors:** Inas Karawia, Mohamed Ashraf Hall, Mona Abdelaziz Elbayoumi, Osama Safwat Mohamed

**Affiliations:** 1 Dental Public Health and Preventive Dentistry, Faculty of Dentistry, Pharos University in Alexandria, Alexandria, Egypt; 2 Alexandria Dental Research Center, Ministry of Health and Population, Alexandria, Egypt; 3 Dental Laboratories Technology, Faculty of Applied Health Science, Masr University for Science and Technology, Giza, Egypt; 4 Dental Laboratory Technology Department, Faculty of Health Sciences at Borg El Arab Technological University, Alexandria, Egypt

**Keywords:** Alkaline water, Chlorhexidine, Streptococcus, Dental caries, Mixed dentition, Diseases, Health care, Medical research, Microbiology

## Abstract

Dental caries is a major public health concern among children, with Streptococcus species, particularly Streptococcus mutans, playing a key role in its development. This study aimed to compare the effect of alkaline water, chlorhexidine mouthwash, and tap water on the salivary Streptococcus count in children aged 10–12 years. A single-blinded preliminary clinical study was conducted on 60 healthy children aged 10–12 years. Three groups of participants were randomly assigned: one group used alkaline water, another group used mouthwash containing 0.12% chlorhexidine, and the third group used tap water as a control. Participants were followed for three weeks. Children with systemic diseases, orthodontic appliances, or recent use of antibiotics or corticosteroids were excluded. Unstimulated saliva samples were collected at baseline and after the intervention. Salivary Streptococcus counts were determined using standard microbiological techniques. After 3 weeks, mean salivary Streptococcus mutans counts (CFU ×10^5^/ml) were 3.0 ± 0.36 in the alkaline water group, 2.1 ± 1.9 in the chlorhexidine group, and 7.9 ± 0.3 in the tap water group. Both intervention groups demonstrated significantly lower bacterial counts compared with the control group (*p* < 0.001), while chlorhexidine showed significantly lower counts than alkaline water. Within the short-term limitations of this study, rinsing with alkaline water demonstrated a significant reduction in salivary Streptococcus mutans counts, although chlorhexidine showed lower bacterial counts after 3 weeks under the conditions of this study. These preliminary findings suggest its potential as an adjunctive oral hygiene agent, and highlight the need for long-term clinical trials to assess its effectiveness in caries prevention.

**Trial registration**: The trial was registered with the Clinical Trial.gov (Number and date NCT06511336, 07/09/2024).

## Introduction

Dental caries is considered one of the major community health concerns, and it is the most widespread chronic disease^1^, with a high prevalence, especially in children^2^. The worldwide prevalence of dental caries among children was reported in 2023 at 46.2% for primary teeth and 53.8% for permanent teeth^3^. This prevalence is related to the different populations, as it is well known that the prevalence of dental caries in developing countries, as well as the Middle East, is high. In Middle East countries (2020), approximately 65% of five-year-old children experience dental caries in their primary teeth. Among 12-year-olds, around 61% are affected in their permanent dentition. The prevalence increases to about 70% by age 15, while the overall rate for children aged 6 to 15 years is estimated to be 66%^4^. In 2019, Abbass et al. reported that the overall prevalence of dental caries in Egypt was approximately 87%^5^.

Dental caries can be inhibited considerably by mechanical and chemical plaque control. Chemical plaque removal can be accomplished using different chemical materials such as fluoride, xylitol, or chlorhexidine^6^. Chlorohexidine is efficient in the elimination of Streptococcus mutans bacteria, which is responsible for the initiation of dental caries^7^. However, chlorhexidine mouthwash has major drawbacks. The most important one, according to Andrucioli et al., who evaluated the effect of chlorhexidine mouthwashes on extrinsic tooth staining, was that chlorhexidine mouthwash used twice a week for 30 days caused clear, extensive tooth staining^8^.

Alkaline water has been proposed as a biologically plausible intervention due to its ability to elevate oral pH and increase oxidation-reduction potential, creating an environment less favorable for acidogenic and anaerobic bacteria such as Streptococcus mutans. Recently, alkaline water has become commercially available for daily use^9^.

Alkaline water’s pH is higher than ordinary drinking water, it has been recognized for its useful effect on some systemic diseases such as dyspepsia, diarrhea, and gastrointestinal hyperacidity^9^. Studies have been conducted to evaluate the neutralizing effect of alkaline water in the oral cavity, it was found that alkaline water increases the salivary pH^10,11^. According to a research in 2019, there was a considerable reduction in salivary colonies after 7 days, indicating that alkaline water can be used as an efficient mouthwash to reduce the quantity of dental plaque bacterial colonies^12^. Furthermore, a study was conducted in 2023 to compare the effect of drinking alkaline water with reverse osmosis water for one week on dental plaque, salivary pH, and salivary Streptococcus mutans count. It was concluded that regular drinking of alkaline water was more effective in reducing plaque and salivary Streptococcus mutans count when compared to reverse osmosis water^13^.

Although previous studies have evaluated the effect of alkaline water on salivary pH and oral microbial parameters, limited evidence is available regarding its short-term effect on salivary Streptococcus mutans levels in children and its exploratory comparison with chlorhexidine mouthwash under standardized conditions. Therefore, the present study aimed to evaluate the short-term microbiological changes associated with alkaline water rinsing in children aged 10–12 years and to compare these findings with chlorhexidine mouthwash and tap water. The null hypothesis of the current study was that no difference would be observed between alkaline water and chlorhexidine mouthwash regarding salivary Streptococcus mutans counts among children aged 10–12 years.

## Materials and methods

The current study was a randomized controlled study, conducted from July 1 to September 30, 2024. Ethical approval was obtained from the ethics committee at Pharos University under the number (UREAC-04-3-222). Written informed consent was obtained from the parents, and the study was registered at ClinicalTrials.gov (PRS) under registration number (NCT06511336) under the link https://clinicaltrials.gov/study/NCT06511336 on 7-9-2024. The study follows the CONSORT guidelines.

### Study participants

The sample size was estimated using G-power software, using 0.69 as the effect size from a previously published study^12^, alpha error 0.05, and power of 0.95. The total sample size was 60 children (20 per group). Participant children aged 10–12 years were recruited from pedodontics clinics in the Faculty of Dentistry, Pharos University, Alexandria, Egypt. Children wearing orthodontic appliances, with systemic diseases, and taking corticosteroids and antibiotics for at least one month were excluded from the study.

### Grouping and intervention (Fig. 1)

The 60 children’s identifiers were written on slips of paper, placed in an opaque container, and randomly allocated by lottery method and equally into 3 groups:

- Group A: rinsing with alkaline water (FLO- bottled natural drinking water, pH 9 - made in Egypt).
- Group B: rinsing with 0.12% chlorhexidine mouthwash (Orovex- MARCO Pharmaceuticals- made in Egypt).
- Group C: rinsing with tap water.

For 3 weeks, children in the 3 groups were instructed to use their mouthwash, according to the assigned group, daily in the evening after teeth brushing with a toothbrush and toothpaste given to the children by the researcher. Children rinsed with 10 ml of mouthwash for 30 s. Parents were given a printed schedule for daily recording to evaluate the children’s compliance in using the mouthwash and toothbrushing.

### Evaluation

Unstimulated 1 ml of saliva was collected by using a plastic syringe for saliva suction^14^ in the morning between 10 and 11 am^15^. The syringes were coded and transported immediately to the microbiological lab in a thioglycolate medium for evaluation by a blinded examiner on the same day. Parents were asked to prohibit their child from eating, drinking anything except water, or brushing their teeth for 2 h before salivary samples collection.

Saliva was collected at baseline, after 1, 2, and 3 weeks.

The saliva samples were collected and immediately transported under strict aseptic conditions to the microbiological laboratory. Upon arrival, the samples were prepared and cultured on selective media, specifically Mitis Salivarius Agar with bacitracin. The plates were then incubated for 7 days at 37 °C in an anaerobic chamber. After incubation, Streptococcus mutans organisms were identified, and the total number of colonies on the plates was digitally counted using a colony counter, with results expressed as CFU/ml.

**Fig. 1:**
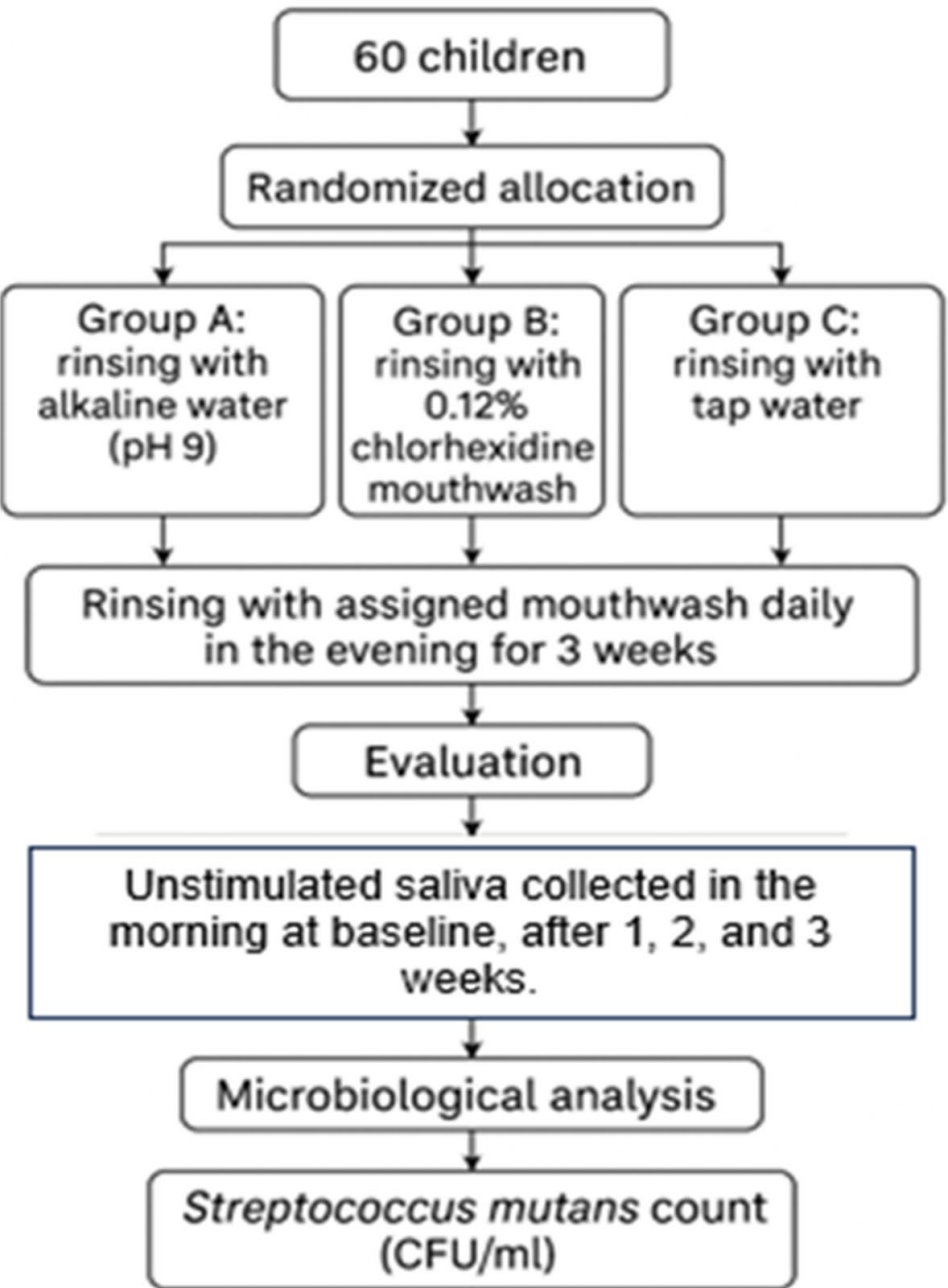
CONSORT flow chart diagram: enrollment, allocation, follow-up and evaluation.

### Statistical analysis

The collected data were reviewed, tabulated, and analyzed using SPSS software, version 25.0. A normality test was conducted, and ANOVA was used to compare the means between groups for quantitative data with a normal distribution. A repeated measures ANOVA was used to assess differences across various follow-up periods, followed by post-hoc tests. The statistical significance level was set at 5% (*p* ≤ 0.05).

## Results

This study was conducted as a single-blinded, randomized, controlled study. A total of 60 participants, half of whom were males, with a mean age of 11.32 ± 1.02 (Table 1), met the inclusion criteria in this study. All participants were equally divided into three groups. No dropout occurred in any groups in this study.

**Table 1:** Distribution of the children by their sex and age.

	No.	%
Sex		
Male	30	50
Female	30	50
Mean age	11.32 ± 1.02	

**Fig. 2:**
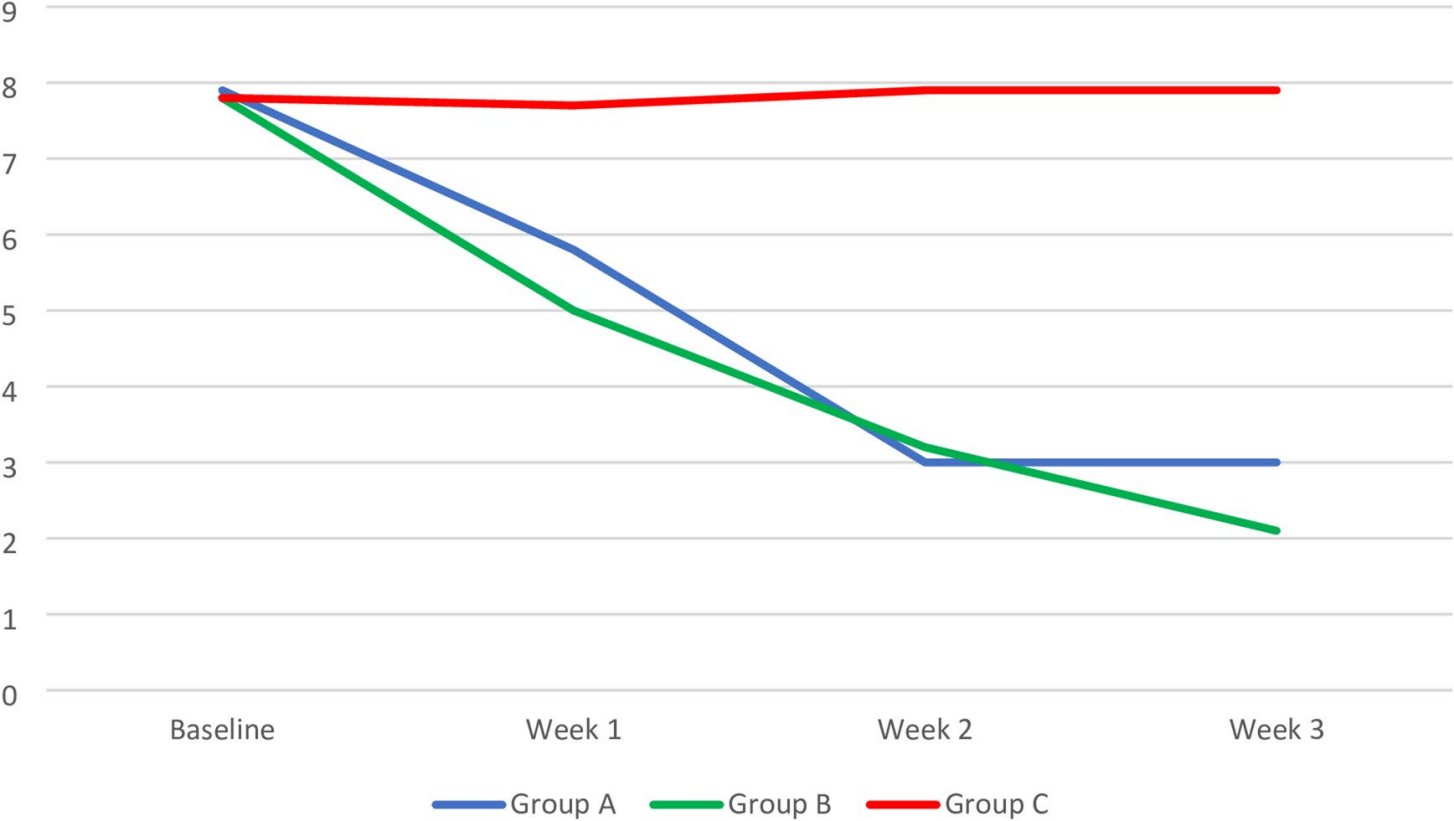
Streptococcus mutans count for different groups in the four follow-up periods.

The distribution of samples in all the groups at baseline, after 1, 2, and 3 weeks, is shown in Table 2; Fig. 2. At **baseline**, all groups had similar Streptococcus mutans counts (mean ± SD ~ 7.8 ± 0.2), indicating uniform initial conditions. **After 1 Week**, *Group A (Alkaline Water)* showed a reduced Streptococcus mutans mean count to 5.8 ± 0.95, *Group B (Chlorhexidine)* also had a reduced Streptococcus mutans mean count to 5.0 ± 0.89, and *Group C (Control)* Streptococcus mutans mean count remained virtually unchanged at 7.7 ± 0.20. The reductions in Groups A and B were statistically significant compared to Group C (*p* = 0.000). **After 2 Weeks**, the Streptococcus mutans mean count in *Groups A* and B was further reduced to 3.0 ± 0.55 and 3.2 ± 1.5, respectively. The mean Streptococcus mutans count in *Group C* slightly increased to 7.9 ± 0.3. Significant differences persisted between intervention groups and control (*p* = 0.000). **After 3 Weeks**, *Group A* showed Streptococcus mutans mean count stabilized at 3.0 ± 0.36, *Group B’s* Streptococcus mutans mean count further decreased to 2.1 ± 1.91 and *Group C’s* Streptococcus mutans mean count remained at 7.9 ± 0.3, showing continued significant reductions in Groups A and B compared to Group C (*p* = 0.000). Chlorhexidine demonstrated significant lower Streptococcus mutans counts than alkaline water after 3 weeks.

**Table 2:** Streptococcus mutans count for different groups in the four follow-up periods.

	Group A CFU x10^5^ mean ± SD	Group B CFUx10^5^ mean ± SD	Group C CFUx10^5^ mean ± SD	*p*-value
Baseline	7.9 ± 0.21^1^	7.8 ± 0.20^1^	7.8 ± 0.22	0.45
After 1 week	5.8 ± 0.95^a2^	5.0 ± 0.89^b2^	7.7 ± 0.20^c^	0.000*
After 2 weeks	3.0 ± 0.55^a3^	3.2 ± 1.5^a3^	7.9 ± 0.3^b^	0.000*
After 3 weeks	3.0 ± 0.36^a3^	2.1 ± 1.9^b4^	7.9 ± 0.3^c^	0.000*
p-value	0.000*	0.000*	0.26	

* Significance level at p value ≤ 0.05.

Different manuscript letters indicate significant differences between groups.

Different manuscript numbers indicate significant differences between different follow-up periods.

## Discussion

Dental caries presents a significant public health challenge, being the most prevalent chronic disease in childhood. Its multifactorial etiology involves a complex interplay of host, diet, and microbial factors. The initiation of dental caries is associated with the activity of Streptococcus mutans. Notably, epidemiological studies consistently demonstrate a direct correlation between a high decayed, missing, and filled teeth (dmft) index in children and elevated counts of Streptococcus mutans. This observation has driven extensive research into various anti-plaque agents, evaluating their efficacy in controlling Streptococcus mutans load^16^. The oral cavity itself represents a highly dynamic and diverse ecosystem, harboring a vast consortium of microorganisms. It is continuously colonized by millions of individual organisms, including more than 250 distinct bacterial species. Within this complex oral microflora, Streptococcus species constitute a particularly essential and abundant component, consistently colonizing both the mucosal membranes and tooth surfaces^17^.

For this study, participants were carefully selected to be between 10 and 12 years of age, a period characterized by mixed dentition. Age is a crucial consideration in subject selection, primarily due to its direct impact on the number of teeth at risk for caries. Furthermore, given that the intervention involved a mouthrinse, selecting children in this age group reduced the potential difficulties younger children might experience with its proper use^18^. The age range of 10–12 years was specifically chosen because these participants are already in a period of heightened caries activity, coinciding with the eruption of permanent teeth^19^.

For microbial analysis in this study, unstimulated saliva was collected using sterile disposable containers. This method provides a representative sample of the basal salivary flow rate^20^.

Streptococcus mutans are known for their ability to develop in environments with high sucrose concentrations and exhibit resistance to the antibiotic bacitracin. This resistance is a key feature exploited by selective media like Mitis Salivarius Bacitracin Agar (MSBA), which is commonly used for their isolation. Accordingly, in this study, Mitis Salivarius Agar was employed as the selective medium for the incubation of salivary Streptococcus mutans. The technique utilized for quantification involved agar plating and subsequent colony counting^21^.

Chlorhexidine was selected as a mouthwash in this context due to its established reputation as the gold standard anti-plaque agent. Its efficacy serves as the benchmark against which other anti-plaque agents are evaluated. The mechanism of action of chlorhexidine is attributed to its cationic effect on plaque, which effectively prevents pellicle formation. Furthermore, chlorhexidine demonstrates a dual antimicrobial action: it is bacteriostatic at lower concentrations^22^ and bactericidal at higher concentrations^20^.

This trial was designed to mimic a realistic at-home regimen, where participants rinsed for 30 s once daily after toothbrushing. The findings of the present study demonstrated a significant reduction in salivary Streptococcus mutans levels with the use of both alkaline and chlorhexidine mouthwashes. These results align with those of Kusumakar et al. (2023), who reported a significant decrease in plaque scores and Streptococcus mutans count after regular consumption of alkaline water (pH 9) at both 7 and 14 days^13^.

The antimicrobial effect of alkaline water can be attributed to several interrelated mechanisms. Primarily, alkaline water elevates the oral pH and enhances salivary buffering capacity, thereby counteracting the acidic environment created by acidogenic and aciduric bacteria such as Streptococcus mutans. Since the initiation and progression of dental caries are highly dependent on sustained low pH, maintaining a more alkaline oral environment disrupts bacterial metabolism and reduces acid production, ultimately inhibiting bacterial growth and virulence^23^.

Additionally, Goyal et al.^24^ proposed that the bactericidal effect of alkaline water may be related to its high negative oxidation-reduction potential, which facilitates increased oxygen availability. This creates an unfavorable environment for anaerobic microorganisms, including Streptococcus mutans, leading to impaired bacterial proliferation. Similar observations have been reported by both Kondo K. et al. and Kurup PR. et al.^10,25^, further supporting the antimicrobial potential of alkaline water.

A study investigated alkaline water on salivary pH in young adults after consuming candy. The study concluded that rinsing with alkaline water after an acidic challenge effectively enhances saliva parameters and neutralizes salivary pH, which can help prevent the caries process in young adults^11^. Similarly, a study at Cebu Doctors’ University aimed to assess how alkaline water affects salivary pH after an acidic challenge from Coca-Cola in students with excellent oral hygiene. The study concluded that alkaline water does affect salivary pH in an acid-challenged oral environment^26^. By stabilizing oral pH above the critical threshold for enamel dissolution, alkaline water may indirectly promote remineralization processes and contribute to caries prevention.

It is crucial to acknowledge that while chlorhexidine (as demonstrated in Group B) continues to be recognized as the gold standard for chemical plaque control, its long-term application is constrained by potential adverse effects. These include dental staining, altered taste perception, and mucosal irritation^27^. Consequently, the utilization of alkaline water presents a potentially gentler and more sustainable alternative to conventional antiseptic agents. This offers an option for individuals aiming to reduce Streptococcus mutans counts and maintain their regular brushing routine, without experiencing the common side effects associated with chemical agents.

### Strengths and limitations

This study has several strengths. It was conducted on a clearly defined pediatric population, with strict inclusion and exclusion criteria to minimize confounding factors such as systemic disease, orthodontic appliance use, and recent antibiotic or corticosteroid intake. Additionally, the inclusion of a control group and a standardized intervention protocol allowed for direct comparison of the effects of alkaline water, chlorhexidine mouthwash, and tap water on salivary Streptococcus mutans counts.

However, several limitations should be acknowledged. The relatively short study duration (three weeks) may not reflect the long-term effects of alkaline water on the oral microbiota or on clinical outcomes such as caries development. In addition, the sample was limited to children aged 10–12 years, which may restrict the generalizability of the findings to other age groups or populations with different oral health profiles. Randomization was performed using a lottery method due to the small sample size, and participant blinding was not feasible because of the use of different branded rinses with distinct sensory characteristics. Nevertheless, the outcome measure was an objective microbiological parameter, and the examiner responsible for bacterial assessment was blinded to group allocation, thereby reducing the potential impact of detection bias.

Furthermore, while the focus on Streptococcus mutans provides insight into a key cariogenic organism, the broader effects of chlorhexidine on the overall oral microbiome were not assessed, and baseline oral hygiene parameters, such as plaque index scores, were not recorded. Although randomization was expected to distribute oral hygiene practices evenly among groups, residual confounding related to differences in brushing effectiveness cannot be completely excluded. Furthermore, salivary Streptococcus mutans counts were used as a surrogate microbiological marker and were not combined with clinical parameters such as plaque indices or caries incidence, which may limit the clinical interpretability of the findings.

Finally, despite standardized instructions, variability in participant compliance with the rinsing protocol cannot be completely excluded. Accordingly, these findings should be interpreted as preliminary and warrant confirmation through well-designed, double-blind randomized controlled trials with larger sample sizes and longer follow-up periods.

## Conclusion

This study showed that rinsing with alkaline water was associated with a significant reduction in salivary Streptococcus mutans counts in children aged 10–12 years over three weeks compared to tap water. However, chlorhexidine demonstrated lower bacterial counts after 3 weeks under the conditions of this study. These findings are preliminary and should be interpreted within the short-term nature and methodological limitations of the study. While alkaline water may represent a potential adjunctive oral hygiene agent with hypothesized advantages, including being a gentler alternative to chlorhexidine, further investigation is required. Double-blind randomized controlled trials with larger and more diverse populations and longer follow-up periods are essential to confirm these effects and assess their clinical relevance for caries prevention.

## Data Availability

The datasets used and/or analyzed during the current study are available from the corresponding author on reasonable request.
